# Sternal Re-Closure for Refractory Dehiscence Using Ultra-High Molecular Weight Polyethylene Tape

**DOI:** 10.1093/icvts/ivag141

**Published:** 2026-05-11

**Authors:** Taiki Niki, Yoshinori Nakahara, Tomohiro Iwakura, Akira Marui

**Affiliations:** Department of Cardiovascular Surgery, Sakakibara Heart Institute, Fuchu, Tokyo 183-0003, Japan; Department of Cardiovascular Surgery, Sakakibara Heart Institute, Fuchu, Tokyo 183-0003, Japan; Department of Cardiovascular Surgery, Sakakibara Heart Institute, Fuchu, Tokyo 183-0003, Japan; Department of Cardiovascular Surgery, Sakakibara Heart Institute, Fuchu, Tokyo 183-0003, Japan

**Keywords:** sternal dehiscence, sternal closure, ultra-high molecular weight polyethylene, coronary artery bypass grafting, reoperation

## Abstract

Ultra-high molecular weight polyethylene tape has been introduced as an alternative to stainless-steel wires for primary sternal closure, yet its efficacy in secondary reconstruction has not been reported. We present a 67-year-old male with refractory sternal dehiscence following coronary artery bypass grafting. Despite 2 failed reconstruction attempts using conventional wires and the Robicsek technique, stable fixation was finally achieved by combining ultra-high molecular weight polyethylene tape with mesh plates. This case highlights the potential utility of ultra-high molecular weight polyethylene tape as a durable and secure option for secondary sternal closure in patients with refractory sternal dehiscence.

## INTRODUCTION

Non-infectious sternal dehiscence is a complication of median sternotomy, and recurrent failure predicts poor prognosis.^1^ Biomechanical studies show that conventional wire closure is vulnerable to dehiscence during coughing^2^ and that alternative fixation methods such as figure-of-eight wiring and band-based systems provide greater rigidity.^3^

The ultra-high molecular weight polyethylene (UHMWPE) tapes, originally developed for orthopaedic use, have recently been adopted for primary sternal closure, with favourable outcomes reported.^4^ However, their use in secondary sternal closure after reconstruction failure has not been reported. Here, we describe a case in which UHMWPE tape achieved definitive sternal stabilization after 2 prior failures.

Informed consent was obtained from the patient.

## CASE PRESENTATION

**Figure 1.**
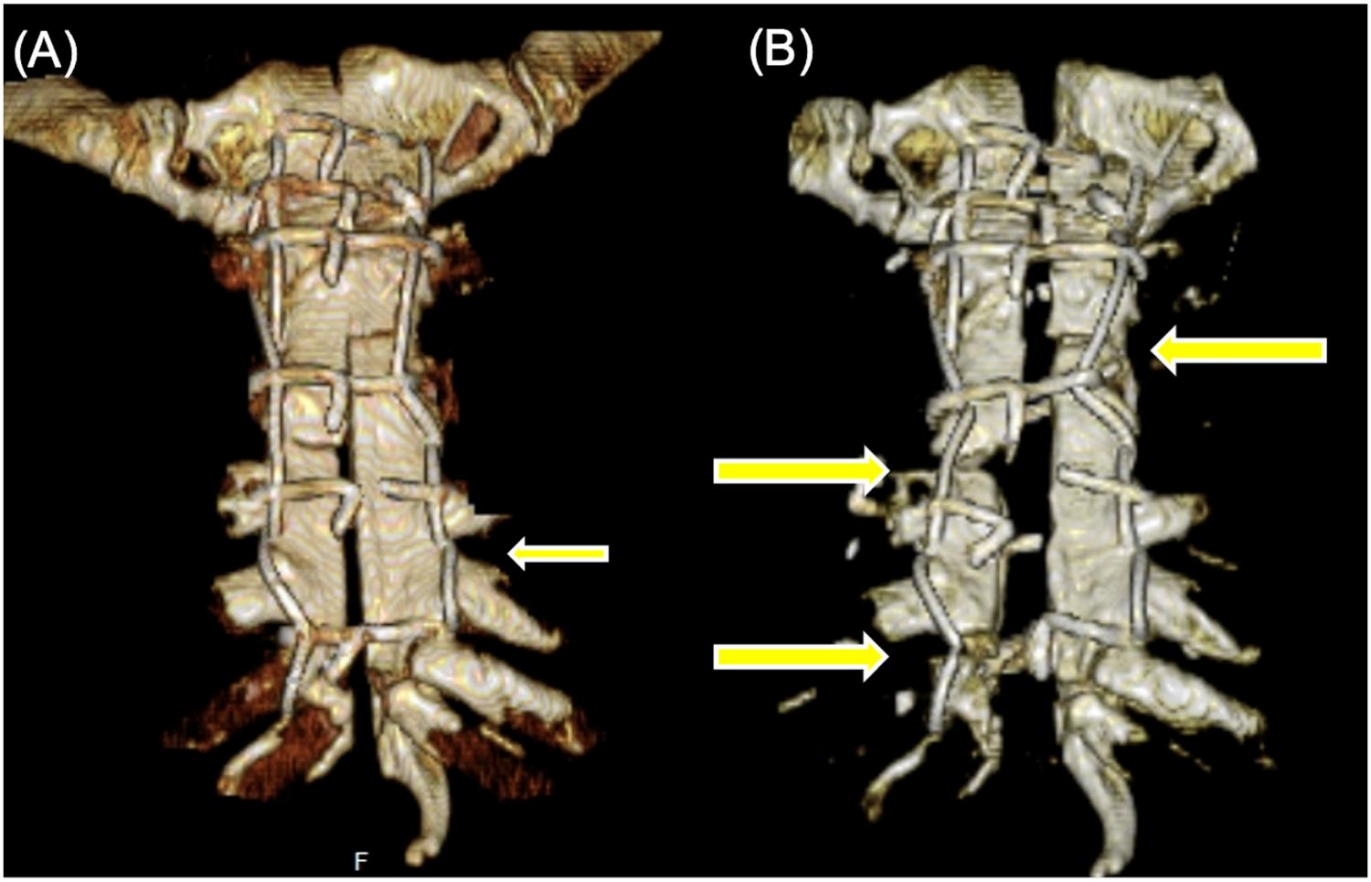
Serial 3 D-CT Images Showing Progression of Sternal Dehiscence. (A) Wire fracture (arrow) and lower sternal dehiscence after initial closure. (B) Progressive sternal fractures (arrows) and worsening dehiscence prior to reoperation.

A 67-year-old male with a history of osteoporosis and bronchial asthma underwent off-pump coronary artery bypass grafting (OPCAB) for exertional angina pectoris. His body mass index was 23.8 kg/m^2^ and his body surface area was 1.67 m^2^. Sternal closure was performed using 6 stainless-steel wires. The postoperative course was complicated by severe coughing due to underlying severe asthma. On postoperative day (POD) 7, the patient experienced intense anterior chest pain following a coughing episode. Computed tomography (CT) conducted on the same day revealed wire fracture and sternal dehiscence (**Figure 1A**). On POD 8, Robicsek re-approximation was performed, and the patient was discharged on POD 20.

Despite pharmacologic interventions and sternal bracing, the cough remained refractory and sternal instability progressively worsened. Follow-up CT conducted at 7 months after OPCAB demonstrated recurrent sternal dehiscence with progressive displacement of the sternal halves (**Figure 1B**).

**Figure 2.**
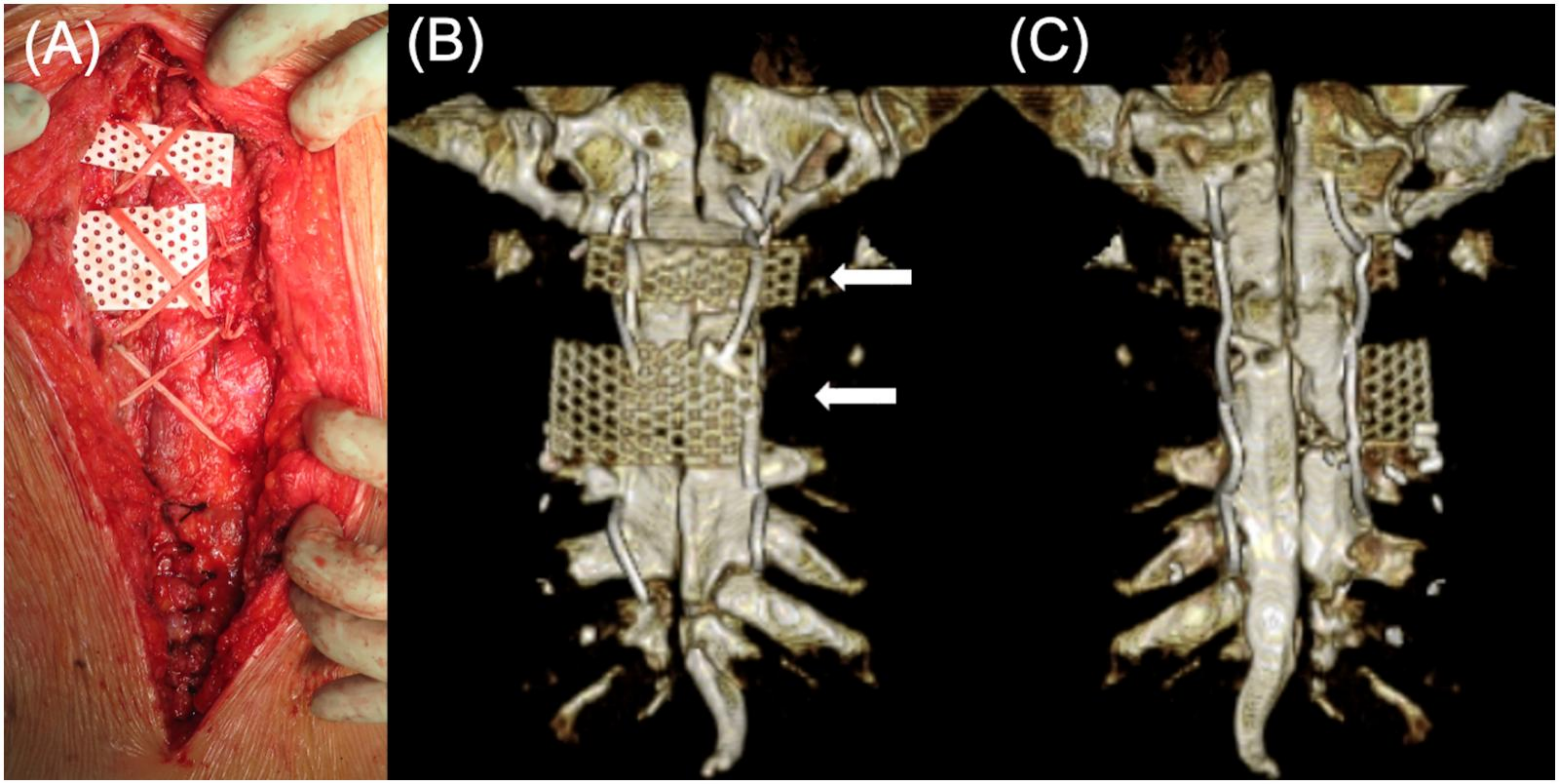
Intraoperative and Postoperative 3 D-CT Findings After Definitive Sternal Reclosure Using Ultra-High Molecular Weight Polyethylene Tape. (A) Intraoperative view showing fractured sternal segments approximated in a figure-of-eight configuration with mesh plates to prevent bone cut-through. (B) Anterior and (C) posterior views of postoperative 3 D-CT demonstrating satisfactory sternal approximation and alignment. White arrows indicate mesh plates.

Eight months after the OPCAB procedure, reoperation for definitive sternal re-closure was performed. Intraoperatively, complete midline sternal dehiscence was confirmed. The circumsternal wires placed during the Robicsek procedure were removed, whereas the lateral reinforcing wires were retained. Both sternal halves exhibited bone fractures. Ultra-high molecular weight polyethylene tapes (NESPLON Cable System, Alfresa Pharma) were placed in a figure-of-eight configuration to approximate the fractured sternal segments, with an additional tape applied at the caudal end. A tape was also placed laterally at the manubrium. Sternal mesh plates were applied to prevent further fracture of the bone fragments (**Figure 2A**).

Postoperative 3 D-CT demonstrated satisfactory approximation and stabilization of the sternal fragments on both anterior and posterior views (**Figure 2B and C**). The patient experienced marked improvement in pain on movement and was discharged on POD 12. At 5 months follow-up, no recurrence of sternal dehiscence was observed.

## DISCUSSION

In this case, repeated wire-based sternal closure resulted in progressive wire fracture and sternal fragmentation. Refractory coughing, osteoporosis, and cumulative sternal substance loss from wire cut-through synergistically contributed to this failure. Ultra-high molecular weight polyethylene tape achieved definitive sternal stabilization in this challenging setting.

Ultra-high molecular weight polyethylene tape has emerged as a promising alternative to conventional stainless-steel wires for sternal closure. It exhibits superior mechanical properties, including higher maximum load and improved fatigue resistance in biomechanical testing.^5^ A calibrated tensioning device enables controlled and reproducible compression, while the broader surface profile of the tape distributes pressure more evenly, minimizing the risk of sternal cut-through and fracture. Favourable outcomes have been reported in high-risk patients undergoing primary sternal closure with this material.^4^ Unlike primary closure, secondary closure presents unique challenges, including fragmented and osteoporotic bone, and loss of sternal substance from prior wire cut-through. The present case demonstrates that UHMWPE tape is also advantageous in secondary re-closure. Although particularly suitable for patients with poor bone quality, active infection is a contraindication owing to biofilm risk within its braided structure. The higher cost may limit routine use, although potential reductions in complications and hospital stay may offset this in high-risk patients.

Mesh plates were applied to provide planar support across the fragmented segments, enabling immediate sternal stabilization after closure. This rigid fixation, together with the broader contact surface of UHMWPE tape, reduced friction between sternal fragments, contributing to prompt pain resolution. The marked pain relief observed in our patient aligns with the findings of Kumar et al.,^4^ who reported significantly lower pain rates and reduced opioid consumption in the suture tape group compared with steel wires.

## CONCLUSION

Ultra-high molecular weight polyethylene tape is a durable option for secondary sternal closure after reconstruction failure, especially when combined with mesh plates. Further clinical data and long-term follow-up are required.

## Data Availability

The data underlying this article are available in the article.
